# Utilising International Statistical Classification of Diseases and Related Health Conditions (ICD)-10 Australian Modification Classifications of “Health Conditions” to Achieve Population Health Surveillance in an Australian Spinal Cord Injury Cohort

**DOI:** 10.1038/s41393-022-00761-6

**Published:** 2022-02-24

**Authors:** Jillian M. Clark, Ruth Marshall

**Affiliations:** 1 Spinal Services, Surgical Specialties, Royal Adelaide Hospital, Adelaide, SA Australia; 2 Centre for Orthopaedics and Trauma Research, The School of Medicine, University of Adelaide, Adelaide, SA Australia; 3 Lifelong Health, South Australian Health and Medical Research Institute, Adelaide, SA Australia; 4 South Australian Spinal Cord Injury Service, Lightsview, SA Australia

**Keywords:** Neurological manifestations, Trauma

## Abstract

**Study design:**

Retrospective, non-randomised, registry controlled.

**Objective:**

To develop a conceptual ICD-10 taxonomic framework for population health surveillance across all-phases of spinal cord injury and disorders (SCI/D).

**Setting:**

Public Hospital Admitted Patient Care (APC) collection, South Australian Dept. Health, South Australia, Australia.

**Methods:**

A core ICD-10-Australian Modification (AM) coded dataset was retrieved from the APC hospital patient admission collection (2012–2017). Search filters and key words referenced to the National Library of Medicine thesaurus identified and quantified incident SCI/D cases. Incident SCI/D case data held in the Australian Spinal Cord Injury Registry (ASCIR) of South Australia (2012–2017) tested fidelity. Data linkage to the South Australian Death Registry controlled for cohort attrition. Both unadjusted and case-mix adjusted core data set yields were evaluated. Outcomes were assessed in terms of APC frequency difference (Δ%) versus ASCIR.

**Results:**

3,504 APC cases were extracted, of which 504 (mean, SD age 55 ± 20 yrs; 348 [69%] male, 202 [39%] traumatic; 135 [32%]) cervical; 51 [10.1%] thoracic and (16 [3.2%]) lumbar met criteria. Comparator data were 385 ASCIR new index cases mean, SD age 56 ± 19 yrs, 229 [75%] male, 162 [42%] traumatic. Case-mix adjusted analysis yielded 336 (APC Δ33%) all-cause incident cases (vs. ASCIR −13 Δ%) and 131 incident cases of traumatic aetiologies (vs. ASCIR −19 Δ%).

**Conclusions:**

The ICD-10 core “Health Condition” data-set assembled extends our understanding of SCI/D epidemiology and with further development may create a cost-efficient and sustainable framework that will improve health system performance and equity within and between countries.

**Sponsorship:**

The Lifetime Support Authority of South Australia sponsored the study.

## Introduction

Spinal cord injuries (SCI) are a heterogeneous group of permanent disabling conditions associated with significant dysfunction involving almost all body tissues and organ systems [1]. A *sine qua non* diagnosis requires three neurological features, motor paralysis, sensory loss and bladder, bowel or sexual dysfunction. The various aetiologies broadly are classified into traumatic (SCI) and non-traumatic syndromes (SCD) of congenital, vascular, neoplastic, pathogenic or toxic causation [1, 2]. These syndromes share common neurological features [1, 2] but can differ substantially in terms of in-hospital mortality risk [3, 4], comorbidity profiles [4, 5] and life expectancy [6, 7].

In consideration of injury surveillance in the context of SCI/D, an important concept in interpreting the epidemiological literature is that differences in the lived experience within or between countries can impart significant differences in prevalence and influence survival, co-morbidity profiles, and the outcomes of survivors [6–9]. At an individual level these differences may or may not involve relationships between demographic, personal, ethno-cultural or socio-economic factors and prevalence or between these factors and the lived experience [6–9]. At the contextual level numerous reports have confirmed that personal^,^ and functional factors, related health conditions, quality of life [9], environmental barriers [8] and socio-economic [10] status may influence the lived experience [11, 12]. Another level of complexity involves the influence of health system performance on these contextual factors and central to this is the interaction between this variable and the quality of public health data repositories or registries. Of relevance to our work, a systematic review uncovered the statistical utility of coded International Classification of Functioning, Disability and Health (ICF)] [13] data for population health surveillance in SCI/D [14]. Extending this conceptual framework, subsequent investigators constructed or went on to validate the psychometric properties of core ICF-10 (WHO, 2001) data-sets coded for five domains; Body Structures and Functions, Activities, Participation, Environmental Factors, and Personal Factors to a diverse range of acute and non-acute [13] clinical outcomes [15–19]. In conjunction with the ICD-10^th^ Edition (1984) [20] classification of “Health Conditions”, the ICF-10 (2001) [14] constitutes the WHO Family of International Classifications (FIC). The key distinction between these FIC members involves their respective sensitivities to classify diseases (ICD-10) [20] and the consequences of diseases (ICF-10) [14]; a concept more generally understood as functional status. We reasoned that the universal acceptance of the core ICD-10 state-of-disease domain “Health Conditions” allowing for statistical comparisons within and between countries may offer an unprecedented opportunity to build a cost-effective and equitable framework for SCI/D disease surveillance.

The development of ICD-10 (WHO, 1984) core state-of-disease datasets with utility for population health surveillance in SCI/D is the subject of our study, which aims to construct a conceptual framework exploiting the ICD-10 coding system of three or four-character rubrics [20] referenced to standardised ICD-10 “inclusive terms.” The data repository selected for analysis is the large ICD-10-AM coded SA Health Public Hospital Admitted Patient Care (APC) collection [21, 22]; also known as the Integrated South Australian Activity Care (ISAAC) collection. The publicly funded APC repository holds codified meta-data for all admitted cases to a public or private hospital in a state-wide jurisdiction accounting for the complete taxonomy of aetiologies of interest to SCI/D epidemiology. Of relevance to the national sociodemographic, the APC repository holds ICD-10-AM alpha and numeric coded data for all admitted episodes of care including acute, subacute and nonacute, with consistency in classification assured by Australian Coding Standards (ACS) developed for use with ICD10-AM and applied in all public and private hospitals nation-wide [22]. Correlates of this experimental rigor are comprehensive cohort coverage and acceptable generalizability [23].

South Australian APC morbidity data are utilised for strategic planning, resource allocation, systems performance measurement and case-mix. An important consequence is their contribution across  the lived experience of this population at the contextual level. Epidemiology in South Australia is the remit of the ASCIR, an opt-in consent registry operated under the mandate of the Australian Institute of Health and Welfare (AIHW), Canberra, Australia [24, 25]. Central to the ASCIR’s contribution to epidemiology is a 25 year-repository of Australian SCI/D annual incident case statistics [24, 25] informing injury prevention, econometrics and clinical research. These relationships have been confirmed for consented cases agreeable to participate in an in-patient rehabilitation programme offered by a specialised public hospital service and the question now is one of whether such findings are generalisable to other settings or an all-cause cohort and sociodemographic. The next step was to realise that systematic thesaurus cross-referencing [28] of SCI/D taxonomic systems [1, 2] to selected, stand-alone ICD-10 “Health Conditions” codes and “inclusive terms” held within the APC repository may be exploited to construct a novel conceptual framework for SCI/D population health surveillance.

In relation to achieving SCI/D population health surveillance the broad study aim was to develop a conceptual ICD-10-AM taxonomic framework to elucidate the distribution and determinants of injury/disease across all phases of SCI/D. To achieve this aim we first evaluated the fidelity of this ICD-10-AM taxonomic framework for point of care identification and quantification of SCI/D cases and secondly, determined the fidelity of this ICD-10-AM taxonomic framework for point of care identification and quantification of new index SCI/D cases. Although of an exploratory nature, we also examined the sensitivity of this stand-alone ICD-10-AM taxonomic framework for point-of-care identification and quantification of new index cases of traumatic aetiology.

The study protocol was approved by the institutional review board of the South Australian Department of Health (Protocol No: HREC/18/SAH/116). We certify that all applicable institutional and governmental regulations concerning the ethical use of data collected for clinical purposes were followed during this research.

## Methods

### Cohorts

The APC cohort selected for study had a private or public hospital admission recorded between Jan 1st, 2012 and Dec 31st, 2017 inclusive and was assigned a WHO ICD-10-AM/ACS code. The codes selected for study included the first 3–4 rubrics (1 alpha and 2–3 numeric) between G82-G83.4 (diseases of the nervous system) and S12-14.1 or 14.10-34.1 (injury poisoning and certain other consequences of external causes) (Table S4). The comparator ASCIR cohort had a public hospital admission with a diagnosis of sub-acute spinal cord injury of traumatic or non-traumatic aetiology, inclusive of *cauda equina* syndrome, conus medullaris syndrome, had ISNCSCI classification ASIA Impairment Scale Grade A, B, C or D, neurological level C1 or below, and had consented to ASCIR registration between Jan 1st, 2012 and Dec 31st, 2017 inclusive.

Cases were excluded if they had a primary diagnosis of progressive or remitting/relapsing intraspinal neurology; e.g., multiple sclerosis, or amyotrophic lateral sclerosis; or critical illness neuropathy (G00-81, G89-99), or radiculopathy (S14.2-9), e.g., brachial plexus lesion; or a S code indicating a diagnosis of vertebral fracture(s), dislocation(s), or fracture/dislocation(s) in the absence of intraspinal neurology.

ICD-10-AM core data-sets were constructed from “Health Condition” codes denoting aetiologies involving the vertebral column, spinal cord, or *cauda equine* (Table S4). Traumatic (accidental, surgical) and non-traumatic aetiologies [2] were included in the core data-set (Table S4), as were personal factors (demographics, socio-economic) and contextual factors [21, 22] described in the data dictionary of the SA Health Admitted Patient Care Data Elements, v1 2020.

De-identified data were retrieved from the large SA Health APC data repository (2012–2017). Data then were cleaned and each ICD-10-AM descriptor of “Health Conditions” decoded and manually matched to its controlled “inclusive term” descriptor to create code-descriptor pairs. Core data-sets then were assembled. Table S4 presents the ICD-10 codes held in the APC collection together with each paired “inclusive term”. Key words referenced to the NLM controlled vocabulary [26] were selected to aggregate data into 2 categories; traumatic and non-traumatic. These categories can also be phrased as SCI and SCD classifications [2].Specifically, the aggregated search terms “injury” and “spinal cord” were selected to classify traumatic aetiology. The aggregated search terms “spinal cord” and “vascular myelopathies”, or “spinal cord” and “inflammatory myelopathies”, or “spinal cord” and “neoplastic disorders” were selected to classify non-traumatic aetiologies. Key word searches were performed using the Microsoft Windows Excel “find” function.

### Framework validation

To test the fidelity of this conceptual framework to identify and quantify SCI/D the new index case data of a SCI/D cohort held in the ASCIR collection (2012–2017) were retrieved for secondary analysis. The ASCIR collection holds the data of an Australian (SA/ NT residents or visitors) cohort presenting for inpatient rehabilitation to the South Australian Spinal Cord Injury Service, South Australia with a definitive clinical diagnosis of SCI/D [2]. Radiological imaging and the International Standard for the Neurological Classification of Spinal Cord Injury [ISNCSCI] [2] routinely are applied in this facility to derive a definitive SCI/D diagnosis (*sine qua non*). Briefly, collected variables include demographic and socio-economic variables, causation, aetiology, and injury/ lesion characteristics (injury level and severity stratified into ISNCSCI AIS Grades A-D), functional (Functional Independence Measure) and process data (length of stay).

### Study outcomes

The WHO/FIC ICD10^th^ ED domain of “Health Conditions” Australian Modification 7^th^ ED was chosen to classify cases at each hospital separation. The primary outcome was the fidelity of an unadjusted ICD-10-coded conceptual framework (Table S4) to identify and quantify cases of SCI/D held in the APC collection. The secondary outcome was the fidelity of an APC case-mix adjusted ICD-10 coded conceptual framework to identify and quantify incident SCI/D cases held in this collection. An exploratory outcome was the sensitivity of case-mix adjusted data to identify and quantify new index cases of traumatic or non-traumatic aetiologies. Analysis of Z, W, and Y codes (“activity from which mechanism of injury is caused”) were rejected due to published concerns about data quality [27]. Primary and secondary outcomes were assessed in terms of ICD-10-AM coded unadjusted and case-mix adjusted data yields respectively, expressed as APC as Δ% of ASCIR data yield.

### Ethical considerations

The low and negligible risk protocol No: HREC/18/SAH/116 was approved under waiver of informed consent. Linkage involved person-level linkage across two data-bases, the ASCIR and South Australian Death Registry. Data integration was conducted under AIHW (ASCIR), and Death Registry Data Custodian approval. ASCIR and Death Registry data were provided in identified form. SA/NT DataLink, South Australia had oversight over linkage quality. To ensure secure transfer ASCIR cases were assigned a re-identifiable code. SA/NT Data-link data linkage keys then were issued. Following linkage, codified linked data were returned to a researcher who was blinded to the study protocol. The waiver of consent linkage protocol utilised to screen for confounders was compliant with WHO Harmony Conference principles [28].

### Statistical analysis

**Fig. 1:**
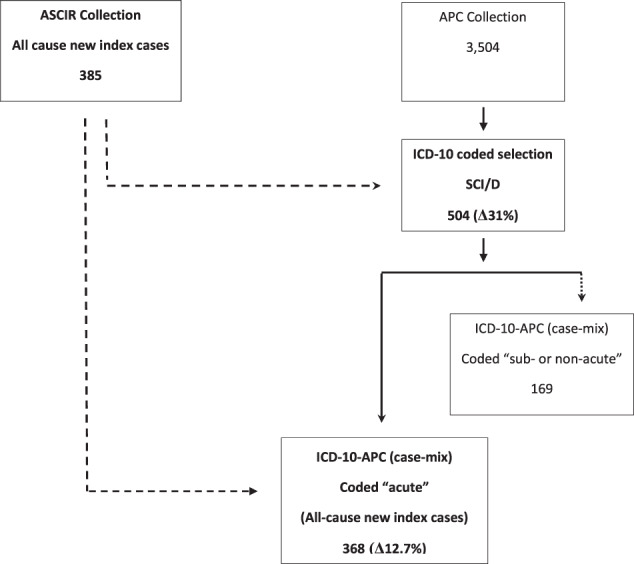
CONSORT flow chart presents filter steps applied to the Australian Admitted Patient Care (APC) repository to test fidelity to detect all-cause new index SCI/D cases (Aim 1). Level 1 = unadjusted APC (R); unadjusted Australian Spinal Cord Injury Registry (ASCIR) (L); Level 2 = International Classification of Disease (ICD-10) adjusted APC (R); and Level 3 = Data Element (case-mix coded “acute”) adjusted ICD-10-APC (R). Data are expressed as: (i) counts (N; solid line); and (ii) APC (N; frequency difference [Δ%]) versus ASCIR (dashed line).

**Fig. 2:**
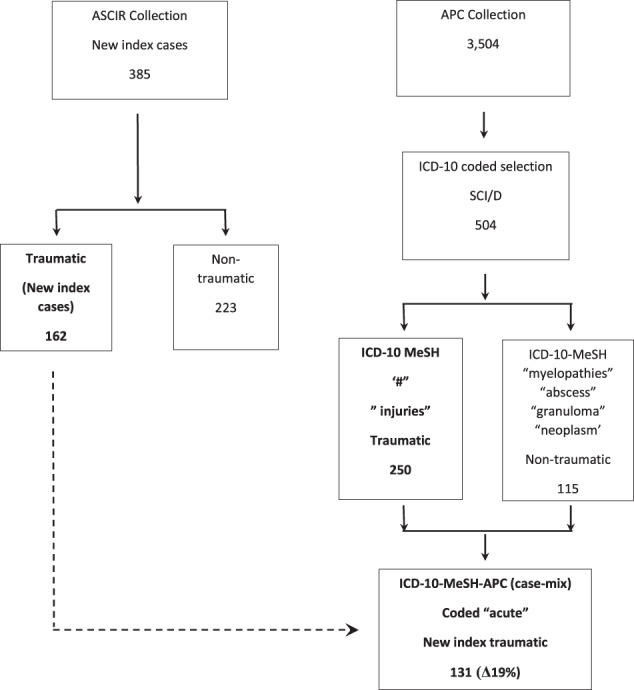
CONSORT flow chart presents filter steps applied to the Australian Admitted Patient Care (APC) and Spinal Cord Injury Registry (ASCIR) repositories to examine fidelity to detect traumatic new index SCI/D cases (Aim 2). Level 1= unadjusted APC (R); Level 2 = International Classification of Disease (ICD-10) adjusted APC (R); and Level 3 = Medical Evidence Subject Heading (MeSH) (“fracture”; “injuries”) adjusted ICD-10-APC (centre-R); Level 4 = Data Element (case-mix coded “acute”) adjusted MeSH-ICD-10-APC (R). ASCIR Level 1= unadjusted ASCIR (L); Level 2= adjusted ASCIR (“traumatic”) (far-L). Data are expressed as: i) counts (N; solid line); and ii) APC (N; frequency difference [Δ%]) versus ASCIR (dashed line).

APC coded data were extracted by codes selected from the ICD-10 “Health conditions”, the data dictionary for this study. Data aggregated by selected ICD-10 code descriptors then were filtered using pre-selected key word search terms. Blank cells were treated as “missing completely at random” and a list-wise deletion strategy applied to remove incomplete cases. CONSORT flow charts (Figs. 1, 2) show the sequence of filtered searches applied to: first, unadjusted and secondly, APC case-mixed adjusted ICD-10 data.

Simple descriptive statistics were applied to parametric variables (means, SD). Categorical variables are reported as frequencies (counts/ percentages). An alpha value of <5% was taken as statistically significant.

### Hypotheses

The a priori hypothesis was: If fidelity to detect new index cases is false then analysis of case-mix adjusted data yield in the extracted APC-ICD-10 (2012–2017) and ASCIR (2012–2017) collections will reveal a frequency difference of > Δ20%. The cut-off point set to reject the null hypothesis was < Δ20%. In the absence of a priori data, effect sizes were calculated from expert knowledge and the SASCIS 2012–2017 referral history (RM).

The secondary hypothesis was: If fidelity to detect new index traumatic cases is false then analysis of case-mix adjusted trauma and acute data yield in the APC and ASCIR collections will reveal a frequency difference of > Δ20%. The cut-off point chosen to reject the null hypothesis for frequency difference in data yield was set at < Δ20%. In the absence of a priori data, effect sizes were calculated from expert knowledge of rehabilitation point-of-care (RM) and acute point-of-care referral data (JMC).

### Power for analysis

Hypotheses were tested using the formula:

APC (N) – ASCIR (N)/ASCIR (N) x100 = Δ%

Analyses were performed using Microsoft Windows 10 Excel software.

## Results

### APC data repository

A large de-identified cohort (3504) of public and private hospital admissions was retrieved from the APC collection (2012–2017). Of this 6-year cohort a subset of 504 cases had an ICD-10-AM code indicative of a *sine qua non* or *forme fruste* SCI/D aetiology, satisfied data integrity criteria, and were retained for analysis.

### Socio-demographic characteristics

Table 1 presents the socio-demographic characteristics of this 6-year ICD-10-AM coded APC cohort. Most identified cases were male (60%), mean cohort age was 53 ± 21 years; with a wide variance. The difference between the mean (SD) age of male and female cases did not reach significance (*p* > 0.05). In relation to ethnicity 25 (5.0%) of the cohort was recorded as Indigenous, as per the NHMRC definition. In terms of family-social support, almost 50% were either married or living in a *de facto* relationship. At the time of admission 144 (29%) cases were engaged in employment. However, a majority had a “not applicable” employment code denoting self-employment, self-funded retiree or benefit recipient. Analysis by type of social benefit revealed that 108 (21%) were in receipt of a benefit payment; with a minor subset of 25 (5.0%) claiming a disability benefit.

**Table 1:** Socio-demographic characteristics of 6-year International Statistical Classification of Disease and Related Health Conditions-10th ED-Australian modification-7TH ED core set of cases.

ICF-10-AM7TH EDN coded cases	*N*	%
Extracted cases (N[%])	504	[100]
Gender		
Male (M; N[%])	348	[69]
Female (F; N[%])	156	[31]
Totals (N[%])	504	[100]
Age at admission		
All cases; yrs (means/SD)	53	21
Males; yrs (means/SD)	55*	20
Females; yrs(means/SD)	57*	21
Ethnicity		
Indigenous (N[%])	25	[5.0]
Non indigenous (N[%])	466	[92]
Not stated (N[%])	13	[2.6]
Totals (N[%])	504	[100]
Marital status		
Married (N[/%])	249	[49]
Never married (N[/%])	142	[28]
Widowed (N/%)	23	[4.6]
Divorced/ Separated (N/%])	27	[5.4]
Status unknown (N[%])	63	[13]
Totals (N[%])	504	[100]
Employment status		
Employed (N[%])	144	[29]
Unemployed (N[%])	28	[5.6]
Student (N[%])	6	[1.2]
Home duties (N[%])	6	[1.2]
NA (N[%])	208	[41]
Other (N[%])	95	[19]
Unknown (N[%])	17	[3.4]
Totals (N[%])	504	[100]
Social Benefits		
Disability (N[%])	25	[5.0]
Age (N,[%])	65	[13]
Unemployment (N[%])	11	[2.2]
Any (N[%])	6	[1.2]
NA (N[%])	217	[43]
Other (N[%])	180	[35]
Totals (N[%])	504	[100]

### Taxonomic framework

Table 2 presents the frequency and distribution of “Health Condition” codes for the 504 APC cases. The most frequently recorded ICD-10-AM code was “Other paralytic syndromes: *Cauda equina* syndrome” (48 [15%]). This was followed closely by coding for “Other diseases of spinal cord: Vascular myelopathies” (46 [14%]).

**Table 2:** International Statistical Classification of Disease and Related Health Conditions-10th ED-Australian Modification-7TH ED cases (=504) ranked by frequency.

ICD-10 “Health Conditions”	Cases (N[%])
Paraplegia and tetraplegia: Paraplegia, unspecified	35 [6.9]
Paraplegia and tetraplegia: Spastic tetraplegia	19 [3.8]
Paraplegia and tetraplegia: Tetraplegia, unspecified	25 [5.0]
Other paralytic syndromes: Cauda equina syndrome	50 [9.9]
Injury of nerves and spinal cord at neck level: Other and unspecified injuries of cervical spinal cord	126 [25]
Injury of nerves and spinal cord at neck level: concussion and oedema of cervical spinal cord: Fracture of the neck unspecified.	9 [1.8]
Injury of nerves and spinal cord at thorax level: Other and unspecified injuries of thoracic spinal cord	51 [10.1]
Injury of nerves and lumbar spinal cord at abdomen, lower back and pelvis level: Concussion and oedema of lumbar spinal cord	10 [2.0]
Injury of nerves and lumbar spinal cord at abdomen, lower back and pelvis level: Injury of cauda equine	6 [1.2]
Fracture of the neck: Fracture of other unspecified cervical vertebrae	20 [4.0]
Fracture of the ribs(s) and thoracic spine: fracture of thoracic vertebrae	18 [3.6]
Fracture of the lumbar spine and pelvis: Fracture of lumbar vertebrae	20 [4.0]
Other diseases of spinal cord: Vascular myelopathies	32 [6.3]
Intracranial and intraspinal abscess and granuloma: Intraspinal abscess and granuloma; extradural and subdural abscess unspecified	29 [5.7]
Other demyelinating diseases of central nervous system: Acute transverse myelitis in demyelinating disease of central nervous system	13 [2.6]
Other diseases of spinal cord: syringomyelia and syringobulbia	5 [1.0]
Inflammatory polyneuropathy: Guillain-Barré syndrome	22 [4.4]
Disorders of autonomic nervous system: Autonomic dysreflexia	6 [1.2]
Malignant neoplasm of spinal cord, cranial nerves and other parts of central nervous system: Spinal cord	8 [1.6]
Totals (N[%])	504 (100)

### Traumatic aetiologies

In terms of traumatic cases, an APC subset of 202 (39%) cases had ICD-10-AM codes referencing the key terms “injury” and “spinal cord”. These terms were taken to indicate a definitive diagnosis of a traumatic SCI or a spinal cord or *cauda equina* syndrome [2]. In rank order, cervical cases were the most prevalent cause of SCI (135 [32%]) followed by thoracic (51 [10.1%]), and lumbar cases (16 [3.2%]). Table 2 presents the distribution and frequency of those traumatic cases identified and subsequently quantitated by ICD-10-AM code.

### Non-traumatic aetiologies

ICD-10-AM codes identifying SCD epochs of care accounted for 115 [23%] of the 504 cases. Of these “vascular myelopathies”, and “intraspinal abscess and granuloma” 28 [8.7%] were most frequently recorded. In terms of omissions, ethical constraints precluded the reporting of rare spinal or spinal cord aetiologies with <5 recorded cases. Due to these constraints the cohort selected for analysis excluded ICD-10 codes for: (i) “Benign neoplasm of meninges: Spinal meninges”; and (ii) “Benign neoplasm of brain and other parts of central nervous system: Spinal cord.”

Table 2 also presents ICD-10 codes for “Guillain-Barré syndrome” (22 cases). Of note, GB is a *forme fruste* diagnosis, and as such new index cases are; first, admitted for inpatient rehabilitation in our facility and secondly, consented to participate and recorded in the ASCIR. For the purpose of this study GB cases were retained for analysis.

### Vertebral column injuries

The NLM descriptors/ search terms “fracture” and “neck”, or “cervical spine”, “thoracic spine” or “lumbar spine” and “fracture” were used in this study to define an epoch of inpatient care due to accidental (low or high impact trauma) or pathological fracture (data not shown). However, from these descriptors it is not reasonable to infer a *sine qua non* diagnosis of SCI/D. Radiological evidence of spinal canal compromise with concomitant spinal cord compression, together with an ISNCSCI physical examination would be necessary to be confident of *sine qua non*. These filters were rejected in the construction of the CONSORT framework (Fig. 1) but may inform future attempts to construct a spinal disorders registry.

### Level of injury (1)

Similarly, inference about level of injury can be extrapolated from the ICD-10 descriptor “tetraplegia or paraplegia unspecified” but this controlled vocabulary cannot distinguish aetiology (e.g., SCI from SCD). Thus, we report that it is not reasonable to derive a definitive SCI/D aetiology or assign an injury level from this single ICD-10-AM 7th EDN code. For reasons involving false positives or false negatives, this filter was rejected and is omitted from Fig. 1.

### Systematic search strategy

To reduce the assumptions introduced by single filters or NLM search terms, we next referenced aggregated ICD-10 codes to the consensus taxonomies of SCD [1] and the ISNCSCI standard [2].

### Level of injury (2)

**Fig. 3:**
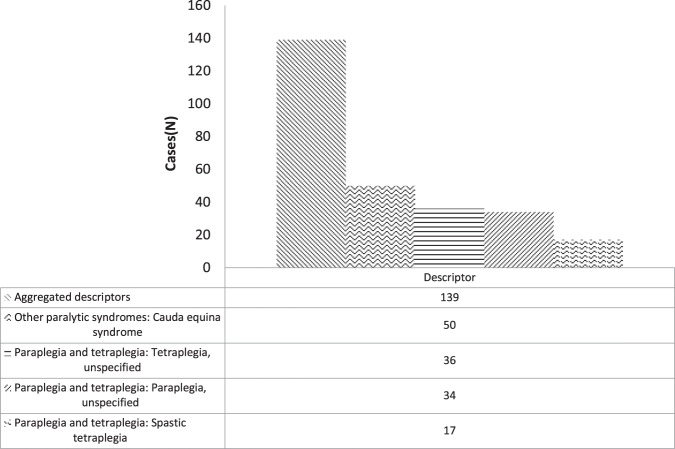
SA Health Admitted Patient Care (APC) – International Statistical Classification of Disease and Related Health Conditions 10th ED (ICD-10) codified data filtered by Medical Evidence Subject Heading (MeSH) search terms (“paraplegia”, “tetraplegia”, “cauda equine syndrome”). Filtered APC-ICD-10 data are presented as frequency distribution (N).

A Windows Excel find function search for all descriptors containing the NLM terms “paraplegia” or “tetraplegia” or “*cauda equina* syndrome” yielded a definitive cohort of 140 (28%) cases (Fig. 3). In rank order, a principal diagnosis of “Other paralytic syndromes: *Cauda equina* syndrome” (50 cases; 9.9%) was the most common followed by “Paraplegia and tetraplegia: Tetraplegia, unspecified” (36 cases; 7.1%) and “Paraplegia and tetraplegia: Paraplegia, unspecified” (34 cases; 6.7%). From this we surmise that when referenced to ICD-10 standards and the controlled NLM vocabulary, an agnostic systematic search strategy can provide some information of relevance to health service planning about level of injury (constrained to 3 categories); with the *caveat* that it is not possible to rule out false positives/ negatives or achieve a precise SCI/D taxonomic classification [1, 2].

**Fig. 4:**
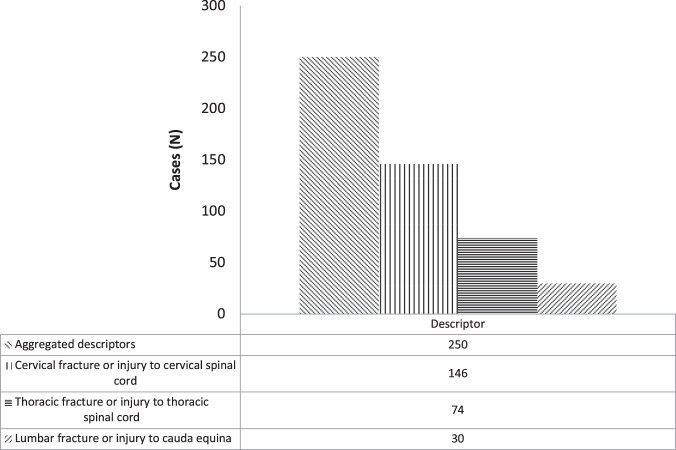
SA Health Admitted Patient Care (APC)-International Statistical Classification of Disease and Related Health Conditions 10th ED (ICD-10) codified data filtered by Medical Evidence Subject Heading (MeSH) search terms (“injury”, “fracture”). Filtered APC-ICD-10 data are presented as frequency distribution (N).

### Acute trauma

Aggregation of broad ICD-10 descriptors and NLM search terms “injury” and “fracture” returned 250 cases (Fig. 4), representing 50% of the cases recorded in the APC collection. This result demonstrates that NLM terms show acceptable accuracy to identify acute trauma admissions in ICD-10 coded data collections; and provide statistical information about the frequency and distribution of damage to body structures and tissues. However, such descriptors / terms cannot distinguish *forme fruste* aetiologies.

In relation to non-traumatic SCD an Excel find search using the aggregated ICD-10 descriptors and NLM search terms “spinal cord” and “vascular myelopathies”, or “inflammatory myelopathies”, or “neoplastic disorders” yielded a subset of 115 (23%) cases. In rank order, a principal diagnosis of “vascular myelopathy” was the most common cause of non-traumatic SCD (32 [6.3%]), closely followed by “spinal abscess” (29 [5.7%]). Extrapolating from ICD-10 descriptors of principal diagnoses (Table 2) it can be deduced that an agnostic search strategy, if referenced to the NLM thesaurus, has some utility to identify non-traumatic SCD cases.

### Case-mix data

**Fig. 5:**
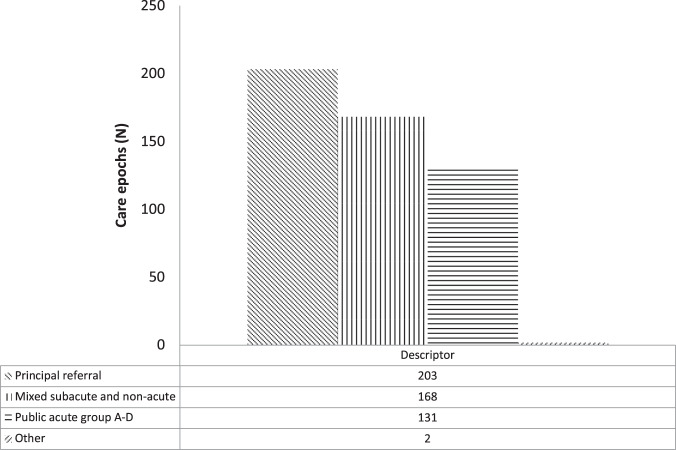
SA Health Admitted Patient Care (APC)-International Statistical Classification of Disease and Related Health Conditions 10th ED (ICD-10) codified data filtered by APC Data Element admission case-mix code (“acute, “subacute”, non-acute”). Filtered APC-ICD-10 epoch of care data are presented as frequency distribution (N).

**Fig. 6:**
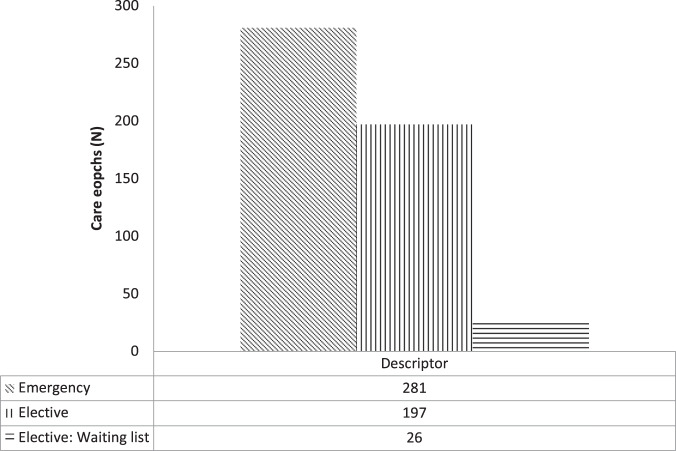
SA Health Admitted Patient Care (APC)-International Statistical Classification of Disease and Related Health Conditions 10th ED (ICD-10) codified data filtered by APC Data Element admission case-mix codes (“emergency”, “elective”). Filtered APCICD-10 epoch of care data are presented as frequency distribution (N).

**Fig. 7:**
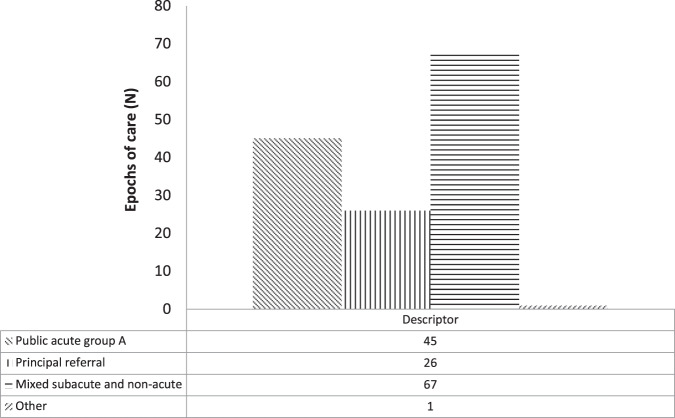
SA Health Admitted Patient Care (APC)-International Statistical Classification of Disease and Related Health Conditions 10th ED (ICD-10) codified data filtered by APC Data Element admission case-mix codes (“public acute”, "principal referral", “mixed subacute or non-acute”). Filtered APC-ICD-10 epoch of care data are presented as frequency distribution (N).

Case-mix data are distributed by SA Health Admitted Patient Care Data Elements admission code. Analysis of the APC data-set by codified case-mix data revealed that 131 (26%) of extracted cases were categorised as “public acute” (Fig. 5). Of the remainder 168 (33%) of extracted cases were classified as “subacute or non-acute” and 203 (40%) as “principal referral” or “other” (2; 0.3%). Case-mix data are presented in Figs. 5–7.

Of these cases, a majority were coded “emergency” (281; 56%), the remainder being “elective” admissions or admissions from an “elective wait list” (Fig. 6). From this, it might be inferred that a strong correlation will exist between the 281 epochs of emergency admission and accidental trauma, and between accidental trauma epochs and ASCIR registered new index cases of traumatic SCI in data adjusted for “vertebral column injuries”.

Fig 3 presents ICD-10 descriptors containing the aggregated terms: “tetra”, “paraplegia” or “*cauda equina*” (refer to Fig. 7) adjusted for APC case-mix. For this subset of 139 cases the mixed “subacute and non-acute” admission code was the most frequently recorded (67 epochs of care); followed by “public acute” (45 epochs of care). In this repository, mixed “subacute and non-acute” admission epochs may indicate an up-transfer to the tertiary hospital from a rehabilitation or another public care facility or an emergency or elective readmission from the community. Thus, subgroup analysis of APC descriptors aggregated by NML terms may be utilised to draw some meaningful conclusions about morbidity across all phases of SCI/D (Table 1).

### New index cases—fidelity to the comparator data repository

The extracted data of 385 new index SCI/D cases recorded on the ASCIR Registry (mean, SD age 56 ± 19 yrs, male 229 [75%] 54 ± 18 y, 18 [4.7%] First Nation, 162 traumatic) were included in this analysis (Table S5). ASCIR-SA Death Registry data linkage uncovered 27 in-hospital deaths, representing 5.4% of the APC epochs of care recorded and 7% of ASCIR case registrations. Because each death was recorded within 6-years post injury, this can be extrapolated to a 90.5% > 6-year survival rate in registered cases. Non-survivors were older (70.5 ± 16 yrs) than survivors (61 ± 19 yrs; *p* = 0.004) and most non-survivors and survivors were male (Table S5). “Neoplastic syndromes” (11, 42.8%) were the most frequently recorded cause of death followed by “sepsis” (8, 28.6%) and “other” categories (8, 28.6%). Overall, 16 (59.3%) primary or secondary causes of death were attributable to a “neoplastic syndrome.” Adjustment of the APC (504) sample for post-acute in-hospital deaths yielded a residual sample of 477, representing and 5% lower level cut-off point for cohort attrition.

Regarding all-cause SCI/D cases, analysis of the frequency difference between unadjusted APC (504) and ASCIR (385) data yield revealed a substantial frequency difference (Δ31%). Of the 504 APC cases 168 had case-mix codes for “sub-acute and non-acute” conditions (Fig. 5). After extraction of these case-mix data 336 (Δ33%) APC cases were retained for analysis (Fig. 2). In this analysis, all ASCIR new index cases were taken as “sub-acute.” Analyses of frequency differences between case-mix adjusted APC data and ASCIR cases returned a modest value of Δ12.7% (Fig. 2). This result satisfied the <Δ20% cut off-point set for the primary outcome to reject the null hypothesis (Aim 1).

In relation to traumatic cases, analysis of APC data coded “fracture or injuries” by the NLM search terms “acute” and “sub-acute or non-acute” yielded 131 “acute” cases. The frequency difference between ASCIR traumatic new index cases and APC “injury/ acute” search terms was Δ19.3% (Fig. 2). Fig 6 presents the distribution of APC case-mix codes “emergency” and “elective” and Fig. 7 ICD codes “paraplegia”, “tetraplegia” or “*cauda equina* syndrome” (Fig. 5) aggregated by case-mix code “acute” or “subacute or non-acute”. “Elective” or “subacute or nonacute” epochs of cares may correlate to up-or down transfers of an in-patient cohort or ambulatory readmissions.

This result confirms the utility of the APC case-mix vocabulary to separate incident from non-incident cases (Aim 2). A *caveat* involves case-mix terms and filters for subacute and nonacute which have no tested sensitivity to distinguish “post-acute” [14] or “longer-term” [19] phases of injury. Our results suggest that refining search terms to a minimum of three and filters to a minimum of two may show superior sensitivity to single terms or individual filters in terms of identifying and quantifying a new incident case of SCI/D (Aim 2).

## Discussion

Referencing of ICD-10 “Health condition” codes to taxonomic concepts was used in this study to construct a conceptual framework for SCI/D population health surveillance. Tools integral to the construction of this ICD-10/FIC based framework were the controlled NLM vocabulary and a systematic search strategy modelled on a Cochrane CONSORT flow framework [26]. Source data consisted of ICD-10-AM data collated in a large South Australian public health repository and the comparator data on new index cases held in a dedicated, South Australian sub-acute or post-acute point-of-care SCI/D registry. Fidelity was assessed in terms of data quantity expressed as frequency difference and frequency/distribution.

The major findings in relation to data quantity were: Analysis of unadjusted ICD-10-AM data yield in relation to comparator data revealed a substantial increase in frequency difference for agnostic APC all-cause aetiologies versus ASCIR subacute point-of-care aetiologies. Notably, this result contrasted markedly with the analysis of case-mix adjusted “acute” ICD-10 data, which uncovered a very modest decrease in frequency difference for our agnostic APC data versus ASCIR subacute point-of-care registration to confirm proof-of-principle. These results do not appear to indicate a spurious effect of systematic omissions of new index cases in the ASCIR collection and specifically, the omission of non-traumatic cases, or an effect involving spurious duplications in our ICD-10 referenced conceptual framework. As shown by analysis of case-mix adjusted ICD-10 data, explanations for the result observed in unadjusted APC data yield appear to involve many subacute up-transfers and non-acute hospital readmissions, presumably related to SCI/D comorbidity profiles [6, 7]. In support of this interpretation is a large literature describing prevalent subacute medical complications and serious health comorbidities across all phases of SCI/D care [6, 7].Our findings show that a multi-dimensional approach utilising a conceptual framework of case-mix adjusted data filtered and refined sequentially via NLM terms can be utilised to quantify both all-cause and traumatic incident cases.

Regarding ICD-10 new index case data yield evaluated in relation to the sub-acute point-of-care ASCIR comparator data obtained for traumatic cases consented for incident case registration, the frequency decrease that was identified in ICD-10 yield was counterintuitive to the thesis that these comparator data suffered from systematic omissions (e.g., peri-morbid mortality, spontaneous recovery not requiring in-hospital care, private hospital transfers, withholding of consent). Thus, it is reasonable to surmise that this ICD-10 framework may help researchers to evaluate the influence of omission or inclusive bias on the extant evidence. Under-representation of traumatic cases in our ICD-10 model may or may not have involved the accuracy or sensitivity of the codes selected to detect incident cases, or the precision of the controlled NML vocabulary to discriminate *sine qua non* diagnoses. These results appear to rule out confounding variables attributable to the insensitivity or specificity of the tools utilised (ICD-10-AM/NML thesaurus) to differentiate *forme fruste* diagnoses (e.g., injuries to the vertebral column with no or transient neurological signs).

In relation to data quality, the demographic characteristics of the ICD-10 core data-set and comparator cohorts demonstrated acceptable convergence in terms of age and gender distribution (Table S5). Case-mix adjusted ICD-10 data also demonstrated an acceptable level of congruence between each repository for incident cases of traumatic aetiology (Table 2). Regarding data capture and data quality, point-of-care data capture was common to both the comparator and APC collections, with the point of distinction being that case-mix diversity is unique to the APC/ ICD-10 coded collection (Figs. 5–7).

In 2012 the estimated incidence of traumatic SCI reported in the USA was 54 new index cases per 1 mill. of the reference population, with an acute in-hospital mortality statistic of 7.4% [3]. Notably, significant correlations were seen between older age at injury in the USA (21%) and peri-morbid mortality [3]. While it is well accepted that demographics, causation and injury characteristics which impact functioning, health and well-being will vary within and between countries [7–10], secondary interactions between physiological aging, prevalent comorbidities of older age, and immunogenic host responses to trauma, at least in part, might explain this association between older age and peri-morbid mortality [3]. Given published census statistics showing the aging of the Australian reference population, it is reasonable to adjust our comparator subacute point-of-care data to account for an effect of peri-morbid mortality, especially in the medically vulnerable elderly population aged 86-years or over [3]. After adjustment for theoretical assumptions about age-related peri-morbid mortality in the comparator APC data-set the frequency difference observed for total data capture in our ICD-10 model reduced from Δ31% to Δ13%. However, due to a dearth of peri-morbid mortality statistics in new index cases admissions to Australian facilities, it is not possible to confirm this theory. We would propose that an important goal in population health surveillance in the context of subacute point-of-care surveillance would be to develop a data integration model with linkage to Death Registry data to adjust the survivor cohort for peri-morbid mortality and acute or subacute in-hospital deaths. An alternative approach may involve adapting our ICD-10 framework to jointly evaluate incident case disease prevalence and mortality. These data are of crucial importance to the effective management of acute respiratory distress syndrome and serious medical complications in new index admissions.

Large scale cohort studies of early sub-acute [14], post-acute [18] and longer-term [19] phases of SCI/D show that variations in functioning, health and wellbeing are attributable, at least in part, to contextual factors [7–10]. Notably, national registries are a platform for the selection of cohorts enroled in many large, international population health initiatives [7–10]. Our data indicate that an understanding of differences in population health surveillance strategies within or between countries or WHO regions is central to the meaningful interpretation of the epidemiological literature. An exemplar, in relation to a national framework for population health surveillance across all-phases of SCI/D care is the well-funded USA Spinal Cord Injury Model Systems [3]. Reference to Spinal Cord Injury Model System reports published to date [3] shows the seminal importance of a better understanding of the influence of case-mix diversity on decision making in spinal cord injury medicine. In our study, a low cost ICD-10 codified search strategy combined with case-mix data demonstrated superior sensitivity to discriminate all-cause incident cases versus three-four rubric ICD-10 “Health conditions” codes alone. This general principle also held for traumatic cases, with the *caveat* that the modest attrition seen in the adjusted ICD-10 data yields versus point-of-care sub-acute surveillance is counterintuitive to USA reports of 7.5% peri-morbid mortality. The reasons for this disparity are unclear but may relate to differences in contextual factors between these countries. Although contingent on data quality, future investigations of the utility of Z,W and Y codes 9 encoding the activity from which mechanism of injury is derived may improve internal validity [27]. In terms of external consistency, the explanation for this failure of ICD-10 coded point-of-acute care cases to reflect an effect of peri-morbid mortality remains unresolved. Importantly, this challenge to research uptake was not common to traumatic and non-traumatic aetiologies. With respect to neoplastic causes the literature shows that a diagnosis of primary or metastatic tumour represented 12% of all 2012 acute SCI admissions and 19% of all non-traumatic cases to a USA facility [4]. Analysis of the incidence of primary or metastatic tumour diagnoses recorded in the comparator ASCIR (14; 3.6% of all cases) and APC/ICD-10 (8; 1.6% of all cases) data collections revealed comparable disease prevalence after adjustment of ICD-10 data for extracted cases (codes < 5). From our case-mix adjusted ICD-10 data a relatively high external consistency for neoplastic aetiologies (n = 12) might be inferred versus ASCIR without conducting a formal Cronbach’s alpha. However, external consistency to published USA data [4] was not established for this variable for any Australian repository. The reason for this apparent difference in the prevalence of neoplastic in-hospital deaths between well-resourced countries is unclear and shows the importance of evaluating contextual factors in conjunction with aetiological taxonomies.

Of relevance to the reproducibility of the study methodology within or between countries and our registry model it is important to note that the WHO-FIC family of taxonomies utilise controlled categories, terms and descriptors derived from the National Library of Medicine thesaurus. This feature of the ICD-10 public hospital data collection was exploited in our study to first, control for data quantity and quality and secondly, constrain for multiple confounders. Notably, the comparator ASCIR data are extracted manually from the medical record and then transcribed. As such data transcription and collation are not controlled by Australian Coding Standards or indexed to the controlled NLM vocabulary. We acknowledge that systematic limitations may have contributed to statistical inference. Our findings invite the notion that a standardised data dictionary or at least a consensus vocabulary indexed to the NLM thesaurus should be utilised across all SCI/D research settings.

Consistency or reliability is distinct from validity, which is defined as the extent to which an instrument measures what it is intended to measure. Major confounders for external validity were revealed in our analysis of individual ICD-10 descriptors of trauma. As discussed above, single search terms were rejected as it is not reasonable to infer a relationship between “vertebral column injuries” and a *sine qua non* diagnosis of SCI. To overcome this limitation MeSH indexing was exploited to create key word search filters within an agnostic search framework. The agnostic search and data retrieval methodology developed in our study mirrors that utilised globally to perform Cochrane data synthesis [26]. Extrapolating from Cochrane principles [8, 26] controlled vocabularies and filters were utilised to identify both all-cause and traumatic new index cases. By extrapolation these residual data map up-transfers and readmissions, as discussed below. Our agnostic search strategy is counterintuitive to the precepts of a quality registry, which is an indisputable epidemiological tool for the surveillance of both prevalent and rare disease conditions [29]. On the other hand, systematic search strategies have well-accepted roles in medical based evidence [26]. In the context of population health surveillance of SCI/D health conditions, filtered searches partnered with consort flow diagrams show the likely importance of when and how data synthesis might be applied.

A limitation to the development of our ICD-10 conceptual framework for SCI/D population health surveillance is that public health source data were constrained to the single domain of “Health Conditions”. Aside from aetiology, the stand-alone ICD-10 three-four rubric encodes information distributed across three (3) anatomical body regions: cervical, thoracic, and lumbar, and two (2) body compartments; vertebral column and spinal cord/ meninges. These five anatomical descriptors contrast markedly with the data yield of the assessor-rated ISNCSCI gold standard [2, 30] (56 dermatomes each scaled 0-2, 10 myotomes each scaled 0–5 and tested bilaterally, and 3 sacral elements scaled present or absent). It is noteworthy that the data held in the comparator ASCIR repository describe ISNCSCI injury severity, level and the functional independence measure at two subacute epochs of care (rehabilitation admission and separation) [24, 25]. Thus, a major limitation of the stand-alone ICD-10 “state-of-disease” data base is that imprecise information about the dimensions of functioning, participation and activities constrains its end-use. In this context, the addition of the three ICF-10 codes for “Body Structures and Functions,” “Activities” and “Participation” to the framework and consideration of contextual factors may provide more information of relevance to SCI/D health system performance than state-of-disease surveillance alone [13–18]. The development of a framework for the psychometric validation of ICF-10 domains “Body Structures” and “Body Functions” as substitutes for standardised and non-standardised SCI outcome measures was the subject of an earlier study by Post et al. [14]. The authors (ref [14]) linked 19 concepts derived from functional outcome measures to 56 ICF “Body Structures” codes and 56 concepts to 114 ICF “Body Functions” codes. Together with our findings these data suggest the desirability of an expanded case-mix adjusted ICD-10/ICF-10 conceptual framework.

A large literature shows that national registries that attempt to collect morbidity and mortality data on individuals with rare disorders can be challenged by operational limitations and cost restraints [29]. In consideration of reported statistics of 54 new index cases per 1 mill. of the USA reference population, it is reasonable to assert that SCI/D might be considered a relatively rare condition. We would propose that strategies which utilise public health source data to develop a sustainable population health surveillance model can offer a cost effective solution in such contexts. The gaps in population health surveillance identified by this and other studies [29] invite the view that agnostic strategies, and perhaps automated tools or artificial intelligence should be utilised to extract meta-data from large public health collections.

### Omissions and duplications

The new index case registry from which the comparator data were sourced has published 22 consecutive annual reports and is an established mainstay of SCI public health planning in Australia [24, 25]. However, the external validity of the comparator data is constrained by its origins as a new index accidental trauma registry operated under the mandate of the Australian Institute of Health and Welfare, NISU, Canberra, Australia. Systematic omissions in this comparator data base include but are not limited to paediatric cases, incident case non-survivors, individuals who are not accepted for rehabilitation and elective transfers to another facility. Notably, the point-of-care surveillance end-point in this registry is rehabilitation discharge, constraining population health surveillance to the subacute phase of SCI/D. In this context a conceptual framework that exploits ICD-10 codified point-of-care acute source data held in large public health repositories to achieve longitudinal population heath surveillance may afford a means to address gaps in population health surveillance [24, 25].

In relation to post-rehabilitation discharge surveillance and data currency, analysis of case-mix data revealed a subset of 168 “sub-acute and non–acute” coded cases (Fig. 5). It is reasonable to assume that these epochs of care delineate up-transfers to the tertiary facility as well as readmissions of non-acute individuals from the community. To resolve the issue of duplicate admissions, case-mix filters were used in our study to extract ICD-10 new index case data. The “sub-acute and non-acute” data identified are a potentially rich source of information and of direct relevance to our understanding of the distribution and determinants of injury/disease across post-acute phases of SCI/D. An additional and complementary role for these case-mix adjusted post-acute ICD-10 data may involve monitoring of longer-term SCI/D prevalence and outcomes and the delivery of care to community-dwelling cohorts.

## Conclusions

Considerable complexity has been revealed by epidemiological research which shows the influence on all-phases of SCI/D care of variables, such as injury-related and contextual factors. These relationships have been explored in dedicated point-of-care SCI/D registries and the question of interest is whether large public health data collections and perhaps data integration strategies might be used to construct a meaningful population health surveillance framework. The implications of an essential role for the standardised ICD-10 taxonomy and controlled NLM vocabulary in injury surveillance are exemplified in our study by case-mix adjusted ICD-10 analysis. The assembled framework extends our understanding of SCI/D epidemiology and with further development may create a tool that will improve health system performance and create health equity within and between countries.

### Data archiving

De-identified data are available upon request and with permission gained from the Data Custodians of the Public Hospital Admitted Patient Care collection, South Australian Dept. Health, Adelaide, South Australia, Australia or the Australian Spinal Cord Injury Registry, Flinders University, Bedford Park South Australia.

## Supplementary information

Table S4 Table S5, Table S6

RECORD CHECK LIST

## Data Availability

APC data are held at the SA Department of Health, in Adelaide, South Australia. ASCIR data are held at the National Injury Surveillance Unit, Flinders University, Bedford Park, South Australia and the AIHW, Canberra, Australia.
