# Supplementary material for: Utilising International Statistical Classification of Diseases and Related Health Conditions (ICD)-10 Australian Modification Classifications of “Health Conditions” to Achieve Population Health Surveillance in an Australian Spinal Cord Injury Cohort
Source: Spinal Cord. 2022 Feb 24;60(8):746–56. doi: 10.1038/s41393-022-00761-6 (PMC9395265; doi:10.1038/s41393-022-00761-6)
Supplement: Supplementary file 1 — Table S4 Table S5, Table S6 [file 41393_2022_761_MOESM1_ESM.pdf]

TABLE S4: WHO year International Statistical Classification of Disease and Related Health Conditions 10th ED core data-set codes and "inclusiveness terms"

ICD-10 Code	Data Dictionary Descriptor
G82	Paraplegia and tetraplegia
G82.0	Flaccid paraplegia
G82.1	Spastic paraplegia
G82.2	Paraplegia, unspecified
G82.3	Flaccid tetraplegia
G82.4	Spastic tetraplegia
G82.5	Tetraplegia, unspecified
G83.4	Cauda equina syndrome
S12	Fracture of the neck
S12.0	Fracture of first cervical vertebra
S12.2	Fracture of other specified cervical vertebra
S13.0	Traumatic rupture of cervical intervertebral disk
S13.2	Dislocation of other and unspecified parts of neck
S13.4	Sprain and strain of cervical spine
S14	Injury of nerves and spinal cord at neck level
S14.0	Concussion and oedema of cervical spinal cord
S14.1	Other and unspecified injuries of cervical spinal cord
S.14.11	Complete lesion of cord
S.14.12	Central cord syndrome
S17	Crushing injury of neck
S19	Other and unspecified injuries of neck
S22	Fracture of rib(s), sternum and thoracic spine
S22.0	Fracture of thoracic vertebra
S23.1	Dislocation of thoracic vertebra
S24	Injury of nerves and spinal cord at thorax level
S24.0	Concussion and oedema of thoracic spinal cord
S24.1	Other and unspecified injuries of thoracic spinal cord
S28	Crushing injury of thorax and traumatic amputation of part of thorax
S29	Other and unspecified injuries of thorax
S32	Fracture of lumbar spine and pelvis
S32.0	Fracture of lumbar vertebra

S33.1	Dislocation of lumbar vertebra
S34.0	Concussion and oedema of lumbar spinal cord
S34.1	Other injury of lumbar spinal cord

TABLE S5: Demographic and injury characteristics of Admitted Patient Care and Australian Spinal Cord Injury Registry cohorts

**Variable**	**APC ICF-10 (504)**	**ASCIR (385)**	**p value**
Age; yrs (means, SD)	55 (20)	56(19)	>0.05
Age males, yrs (means, SD)	53(21)	*54 (18)	>0.05
Age females, yrs (means, SD)	57 (21)	*59 (20)	>0.05
Male: Female [M:F]	2:1	3:2	-
New index cases [T, NT] (N, %)	368	385	-
New index cases [T], (N, %)	131	162	-
Traumatic: Nontraumatic [NT]	1:2	1:2	-

TABLE S6: Source and comparator data protocols

**Variable**	**APC**	**ASCIR**
Geographic region	South Australia and The Northern Territory	South Australia and The Northern Territory
Observation period	Jan 1st, 2012 and Dec 31st, 2017	Jan 1st, 2012 and Dec 31st, 2017
Ethics approval	SA Health and Well-being	Flinders University
Setting	All public and private hospitals	Specialist spinal cord injury rehabilitation service
Design (Site)	Multiple	Single
Design	Observational, prospective	Observational, prospective
Participation	Waiver of consent	Opt-in consent
Participants	All-cause SCI/D; all epochs of care	All cause SCI/D; single epoch of care eligible for inpatient rehabilitation
Sample points	Hospital Separation	Inpatient rehabilitation admission and separation
Repeat measures	Single or multiple sample points, N (uncontrolled) ≥1	Multiple sample points, N (controlled) = 2
Data format	De-identified	Identified
Data quality	ACS controlled	NISU data cleaning
Inter-observer error	ACS Guidelines	Uncontrolled
Intra-observer error	ACS Guidelines	Uncontrolled
Data dictionary	NLM thesaurus	Registry-based
Data integrity	SA Health	Site - Flinders University
